# The effect of intraperitoneal instillation of drugs on postoperative analgesia after laparoscopic cholecystectomy: a network meta-analysis

**DOI:** 10.3389/fphar.2025.1646917

**Published:** 2025-09-12

**Authors:** Dongmei Zhang, Xiaojiao Wang, Xiaoli Yang, Dajian Xia

**Affiliations:** ^1^ Department of Emergency, The Affiliated Dazu’s Hospital of Chongqing Medical University, Chongqing, China; ^2^ Department of Critical Care Medicine, The Affiliated Dazu’s Hospital of Chongqing Medical University, Chongqing, China

**Keywords:** intraperitoneal, laparoscopic cholecystectomy, postoperative analgesia, drug intervention, network meta-analysis

## Abstract

**Background:**

Postoperative pain is a critical factor contributing to delayed discharge and postoperative recovery after laparoscopic cholecystectomy (LC). Intraperitoneal instillation of analgesic agents has been proposed as a means to alleviate pain in patients undergoing LC. This study aimed to evaluate the efficacy of various drugs administered via intraperitoneal instillation for postoperative analgesia after LC using a network meta-analysis approach.

**Methods:**

A comprehensive search was conducted in PubMed, EMbase, Web of Science and Cochrane Library databases from inception to August, 2025. Randomized controlled trials (RCTs) investigating the effects of intraperitoneal instillation on post-LC analgesia were included. Two independent reviewers screened studies, extracted data, and assessed the risk of bias. A frequentist network meta-analysis was performed to estimate standardized mean differences (SMDs) and 95% confidence intervals (CIs). The surface under the cumulative ranking curve (SUCRA) was used to rank the interventions for each outcome.

**Results:**

Eleven RCTs comprising 667 patients were included. According to SUCRA values, bicarbonate (96.5%) ranked highest in reducing VAS scores at 24 h post-surgery. Acetazolamide (85.9%) was most effective at 12 h, MgSO_4_ (98.4%) at 6 h, and ondansetron (96.4%) at 2 h. Dexamethasone was associated with the lowest analgesic consumption (SUCRA: 95.3%) and the longest time to first analgesic request (81.5%).

**Conclusion:**

Intraperitoneal instillation of bicarbonate, acetazolamide, MgSO_4_, and ondansetron provides differential analgesic benefits at various time points after LC. Dexamethasone appears to be a promising adjunctive agent for reducing analgesic requirements and prolonging the duration of analgesia.

## 1 Introduction

Laparoscopic cholecystectomy (LC) is widely regarded as the first-line treatment for gallstones, polyps, and cholecystitis (Straatman et al., 2023). Offering advantages such as minimal invasiveness, reduced postoperative pain, shorter hospital stays, and a favorable safety profile, LC aligns with the principles of minimally invasive surgery and has become the gold standard for managing these conditions (Wu et al., 2023; Bauiomy et al., 2025). Nevertheless, postoperative pain remains a common clinical challenge, significantly prolonging recovery time and contributing to delayed discharge (Xu et al., 2023; Zhu and Sun, 2023; Cheng et al., 2023). One study reported that 65% of patients experienced moderate pain and 23% reported severe pain within 24 h after LC (Kavanagh et al., 2008). Thus, developing effective analgesic strategies is essential for improving postoperative outcomes in these patients.

Epidural and intrathecal analgesia are established gold standards for pain management in abdominal surgery (Marks et al., 2012). Specifically for LC and gynecological procedures, intraperitoneal instillation of analgesic agents has demonstrated efficacy in reducing postoperative pain (Marks et al., 2012; Abdelhedi et al., 2023). Commonly administered drugs include local anesthetics, corticosteroids, opioids, and α2-adrenergic receptor agonists (Marks et al., 2012; Abdelhedi et al., 2023; Abdelaziz et al., 2021; Beder El Baz and Farahat, 2018; Honca et al., 2014; Labaille et al., 2002; Melidi et al., 2016; Nikoubakht et al., 2022; Putta et al., 2019; Rahimzadeh et al., 2018; Sravanthi et al., 2023; Vijayaraghavalu and Bharthi Sekar, 2021). A meta-analysis by Wei et al. indicated that intraperitoneal levobupivacaine significantly alleviated pain following LC (Wei and Yao, 2020). Similarly, Choi et al. reported that intra-abdominal local anesthesia effectively reduced abdominal, visceral, and shoulder pain at rest in LC patients (Choi et al., 2015). Another meta-analysis by Yong et al. found that ropivacaine instillation not only decreased postoperative pain but also was associated with fewer adverse events compared to controls (Yong and Guang, 2017). These findings support the use of intraperitoneal instillation as a valuable strategy for postoperative analgesia in LC.

Although several systematic reviews and meta-analyses have evaluated specific regimens (Xu et al., 2023; Wei and Yao, 2020; Yong and Guang, 2017), the recent publication of numerous randomized controlled trials (RCTs) (Abdelhedi et al., 2023; Nikoubakht et al., 2022; Sravanthi et al., 2023; Vijayaraghavalu and Bharthi Sekar, 2021) has expanded the evidence base without establishing consensus on the optimal agent. This uncertainty complicates clinical decision-making regarding pain management. To address this gap, we conducted a systematic review and network meta-analysis of available RCTs to comprehensively compare and rank multiple intraperitoneal interventions. Our aim is to provide robust and comprehensive evidence to inform clinical practice in selecting the most effective analgesic protocol for patients undergoing LC.

## 2 Materials and methods

This network meta-analysis was conducted in accordance with the Preferred Reporting Items for Systematic Reviews and Meta-Analyses extension statement for Network Meta-Analyses (PRISMA-NMA) (Hutton et al., 2015).

### 2.1 Search strategy

A comprehensive literature search was performed in the following electronic databases: PubMed, EMbase, Web of Science and Cochrane Library. The search encompassed all publications from database inception up to August, 2025. We aimed to identify all RCTs investigating the effect of intraperitoneal instillation of drugs on postoperative analgesia after LC. To minimize the risk of omitting relevant studies, we also manually screened the reference lists of all included articles and related systematic reviews. The search strategy incorporated both free-text terms and controlled vocabulary (e.g., MeSH and Emtree terms), including but not limited to: “intraperitoneal”, “laparoscopic cholecystectomy”, and “randomized controlled trial”. The detailed search strategy is provided in Supplementary Table S1.

### 2.2 Inclusion and exclusion criteria

Inclusion criteria:

1. Study design:RCTs;
2. Population: patients undergoing LC;
3. Interventions: intraperitoneal instillation of any drug, compared against any other active intervention or placebo;
4. Outcomes: The primary outcome was pain intensity measured using the visual analog scale (VAS) at 24 h postoperatively; Secondary outcomes included VAS scores at 2, 6, and 12 h after surgery, time to first request for analgesic rescue, and total analgesic consumption.

Exclusion criteria:

1. Duplicate publications;
2. Studies with missing or incomplete outcome data;
3. Interventions not relevant to the review question;
4. Non-RCT publications, such as reviews, systematic reviews, conference abstracts, case reports, or commentaries.

### 2.3 Data extraction

All retrieved records were imported into EndNote software for duplicate removal. Two independent reviewers screened the titles and abstracts of studies against the predefined inclusion and exclusion criteria. Potentially eligible articles underwent full-text review. Data were extracted from the included studies using a standardized form, with cross-verification between reviewers. Any discrepancies were resolved through discussion or by consultation with a third reviewer. The following data were collected: first author, year of publication, sample size, age, detailed intervention, body mass index (BMI), outcomes, and results of risk of bias assessment.

### 2.4 Risk of bias assessment

The methodological quality of the included RCTs was evaluated using the Cochrane Risk of Bias Tool (Higgins et al., 2011). Domains assessed included: random sequence generation, allocation concealment, blinding of participants and personnel, blinding of outcome assessment, incomplete outcome data, selective reporting, and other potential sources of bias. Each item was judged as “low risk”, “high risk”, or “unclear risk” of bias.

### 2.5 Statistical analysis

Network meta-analysis was conducted using Stata software (version 14.0). For continuous outcomes, treatment effects were expressed as standardized mean differences (SMDs) with 95% confidence intervals (CIs). Heterogeneity among studies was quantified using the *I* ^*2*^ statistic. An *I* ^*2*^ value below 50% with a corresponding *p* > 0.10 indicated acceptable heterogeneity, and a fixed-effects model was applied; otherwise, a random-effects model was used.

The evidence network was graphically summarized where node sizes represented sample sizes per intervention and edge thickness indicated the number of studies connecting two treatments. If closed loops were present, inconsistency was assessed using node-splitting analysis, which evaluates disagreement between direct and indirect evidence. A *p*-value > 0.05 suggested no significant inconsistency, and a consistency model was adopted. The surface under the cumulative ranking curve (SUCRA) was computed to rank the interventions for each outcome, with higher SUCRA values (0%–100%) indicating better performance. A comparison-adjusted funnel plot was generated to evaluate potential publication bias and small-study effects.

## 3 Results

### 3.1 Literature search results

**FIGURE 1:**
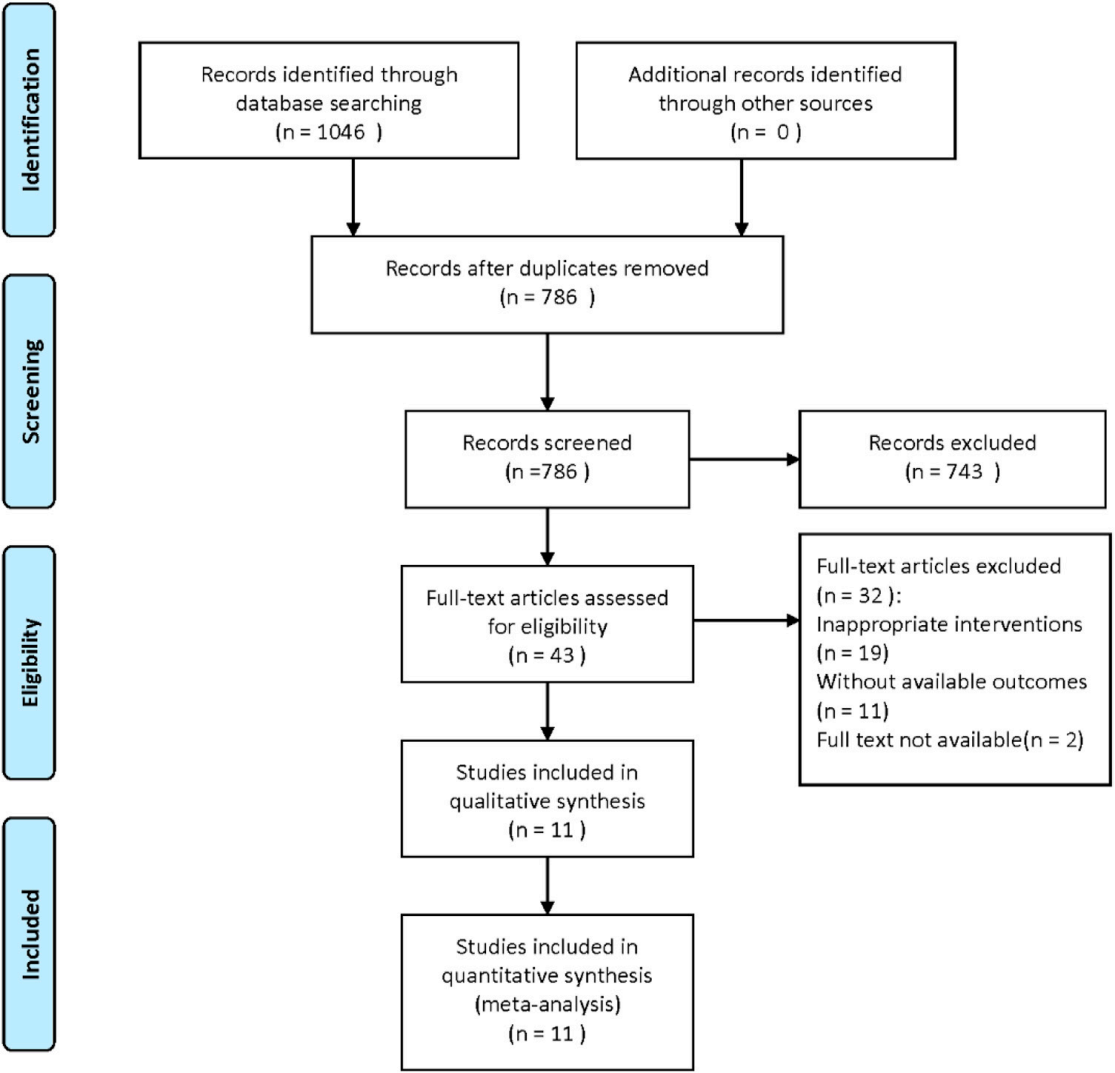
Literature screening flow chart.

A total of 1,046 records were retrieved from the databases. After importing into EndNote, 260 duplicates were removed, resulting in 786 unique articles. Following title and abstract screening, 743 articles were excluded as irrelevant. The full texts of the remaining 43 articles were assessed for eligibility, and ultimately, eleven studies (Abdelhedi et al., 2023; Abdelaziz et al., 2021; Beder El Baz and Farahat, 2018; Honca et al., 2014; Labaille et al., 2002; Melidi et al., 2016; Nikoubakht et al., 2022; Putta et al., 2019; Rahimzadeh et al., 2018; Sravanthi et al., 2023; Vijayaraghavalu and Bharthi Sekar, 2021) were included in the analysis (Figure 1).

### 3.2 Basic characteristics of literature

The eleven included studies involved a total of 667 patients and evaluated nine different drugs. The baseline characteristics of these studies are summarized in Table 1.

**TABLE 1:** Characteristics of the included studies.

References	Stata	ASA	Sample size	Sex (Male/Female)	BMI (kg/m2)	Age (Years)	Interventions
Abdelhedi et al. (2023)	Tunisie	I-II	30	13/17	26.61 ± 3.85	50.97 ± 15.96	Dexamethasone
			30	7/23	27.11 ± 2.54	46.83 ± 10.78	Placebo
Labaille et al. (2002)	France	I-II	14	3/11	NA	53 ± 15	Ropivacaine
			12	4/8	NA	45 ± 18	Placebo
Honca et al. (2014)	Turkey	I-II	30	NA	NA	45.8 ± 14.1	Bupivacaine
			30	NA	NA	47.2 ± 13.2	Levobupivacaine
			30	NA	NA	44.63 ± 9.2	Placebo
Melidi et al. (2016)	Greece	I-II	36	19/17	26.9 ± 2.6	52 ± 13.7	Levobupivacaine
			37	18/19	26.2 ± 4.1	51.24 ± 14.3	Placebo
Nikoubakht et al. (2022)	Iran	I-II	19	5/14	25.65 ± 4.01	44.89 ± 11.41	Bicarbonate
			19	4/15	24.56 ± 2.89	46.84 ± 14.46	Marcaine
			20	5/15	24.49 ± 7.18	41.20 ± 13.38	Placebo
Putta et al. (2019)	India	I-II	28	11/17	25.0 ± 4.0	46.7 ± 8.7	Bupivacaine
			28	13/15	24.2 ± 3.2	41.0 ± 9.0	Placebo
Rahimzadeh et al. (2018)	Iran	I-II	20	NA	28.6 ± 1.8	45.5 ± 6.64	Bupivacaine
			20	NA	28.3 ± 1.9	44.3 ± 5.31	Acetazolamide
			20	NA	27.2 ± 1.9	47 ± 5.64	Placebo
Vijayaraghavalu and Bharthi Sekar (2021)	India	I-II	30	19/11	NA	39.8	Bupivacaine
			30	19/11	NA	40.13	Placebo
Sravanthi et al. (2023)	India	I-II	32	10/22	23.50 ± 2.1	42.59 ± 12.03	MgSO4
			32	12/20	24.25 ± 1.1	42.84 ± 11.32	Placebo
Beder El Baz and Farahat (2018)	Egypt	I-II	35	8/27	29.9 ± 6.9	NA	Levobupivacaine
			35	10/25	31 ± 6.2	NA	Placebo
Abdelaziz et al. (2021)	Egypt	I-II	25	7/18	NA	40.98 ± 15.33	Ondansetron
			25	5/20	NA	37.85 ± 13.17	Placebo

Abbreviation: ASA, american society of anesthesiologists; BMI, body mass index; NA, data Not Available.

### 3.3 Biased risk assessment

**FIGURE 2:**
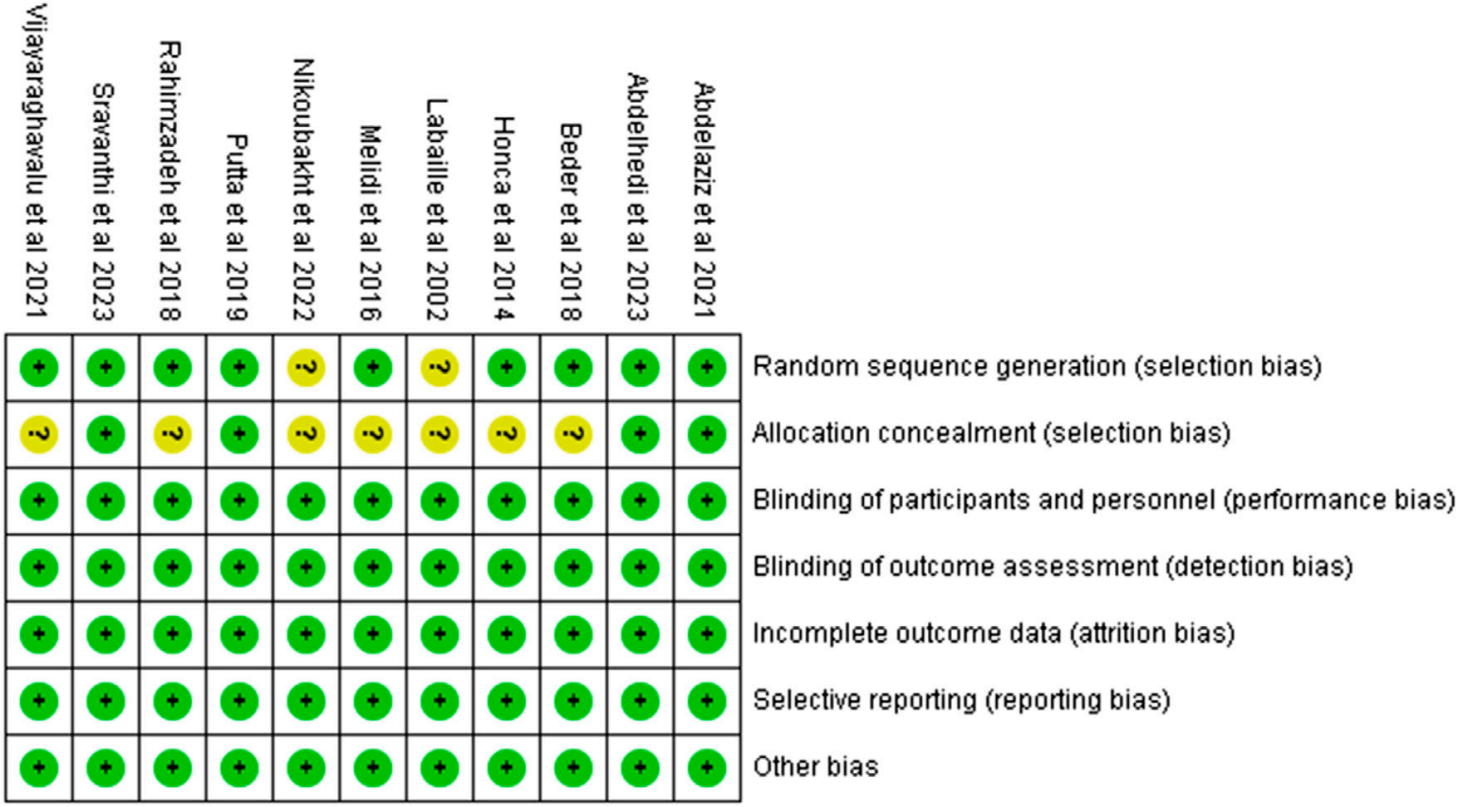
Risk of bias summary.

Two studies exhibited a high risk of bias in the method of randomization. In seven studies, there was some concern regarding risk of bias related to allocation concealment. All other domains were assessed as low risk across the included studies (Figure 2; Supplementary Table S2).

### 3.4 Pairwise meta-analysis

#### 3.4.1 24-h postoperative visual analog scale score

The pairwise meta-analysis indicated that both bupivacaine and levobupivacaine were superior to placebo in reducing VAS scores at 24 h post-surgery. Descriptive analyses further suggested that bupivacaine, dexamethasone, acetazolamide, bicarbonate, marcaine, and ondansetron also outperformed placebo. Additionally, acetazolamide was associated with lower VAS scores than Bupivacaine at this time point. Detailed results of the pairwise comparisons are provided in Supplementary Table S3.

#### 3.4.2 12-h postoperative visual analog scale score

According to the pairwise meta-analysis, levobupivacaine demonstrated a significant reduction in VAS scores compared to placebo at 12 h postoperatively. Descriptive analyses indicated that acetazolamide, dexamethasone, and ondansetron were also more effective than placebo. Moreover, acetazolamide showed superior efficacy to bupivacaine in reducing VAS scores at 12 h. Full results are available in Supplementary Table S3.

#### 3.4.3 6-h postoperative visual analog scale score

The pairwise meta-analysis revealed that both bupivacaine and levobupivacaine were more effective than placebo in lowering VAS scores at 6 h after surgery. Descriptive analyses also indicated that dexamethasone performed better than placebo at this time point. Complete results can be found in Supplementary Table S3.

#### 3.4.4 2-h postoperative visual analog scale score

Based on the pairwise meta-analysis, bupivacaine was associated with significantly lower VAS scores than Placebo at 2 h post-surgery. Descriptive analyses suggested that both bicarbonate and marcaine also outperformed placebo. Furthermore, bicarbonate was superior to marcaine in reducing early postoperative pain at the 2-h mark. See Supplementary Table S3 for detailed results.

#### 3.4.5 Analgesics consumption

Descriptive analyses indicated that both dexamethasone and levobupivacaine led to a significant reduction in analgesic consumption compared to placebo. The results of the pairwise meta-analysis are presented in Supplementary Table S3.

#### 3.4.6 First analgesic requirement time

Descriptive analyses showed that MgSO_4_, levobupivacaine, bupivacaine, and dexamethasone were associated with a longer time to first analgesic request compared to placebo. Additionally, bupivacaine prolonged the time to first analgesic requirement compared to levobupivacaine. Detailed results are available in Supplementary Table S3.

### 3.5 Network evidence plot

**FIGURE 3:**
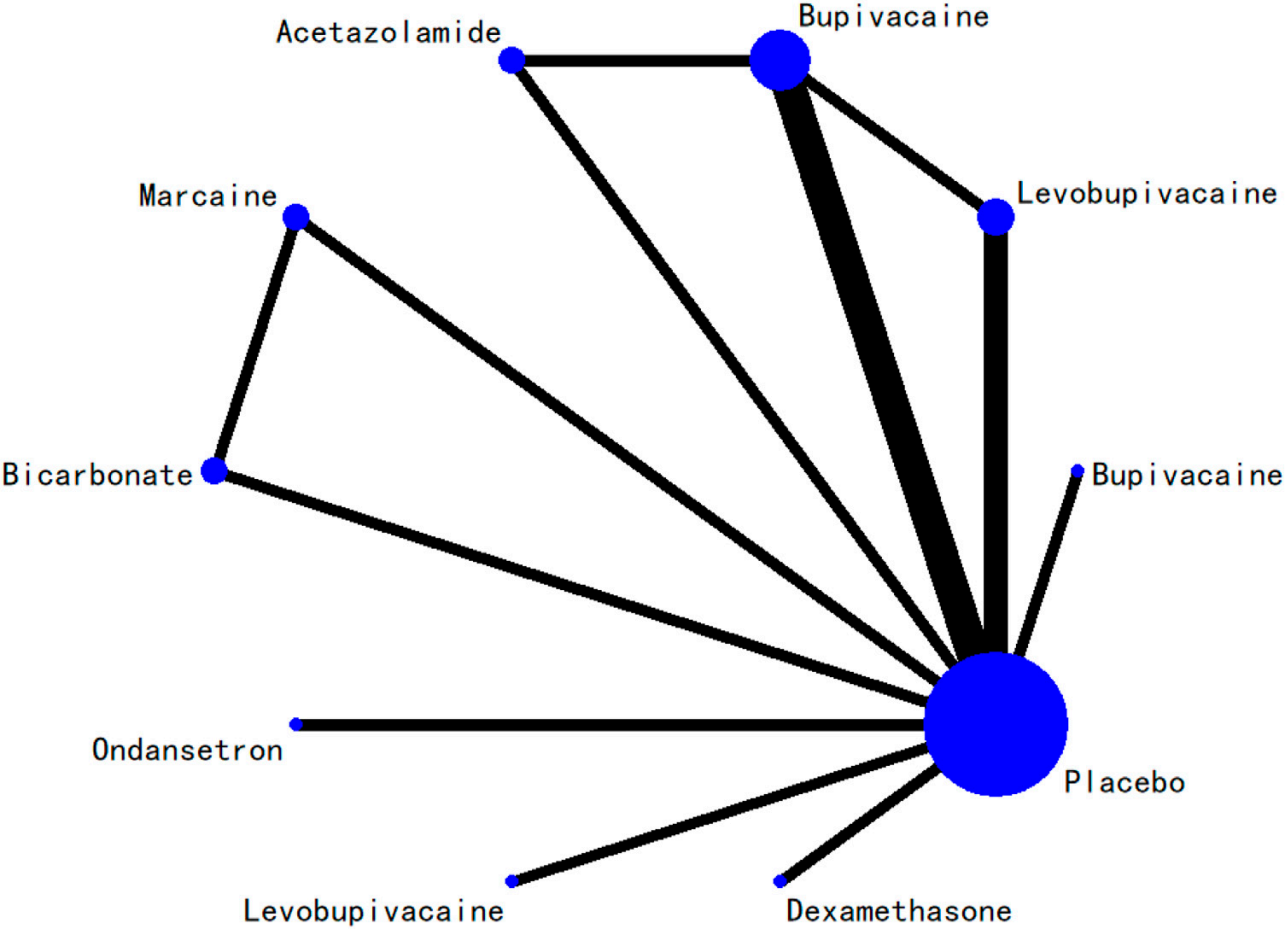
Network evidence diagram for 24-h postoperative visual analog scale score.

**FIGURE 4:**
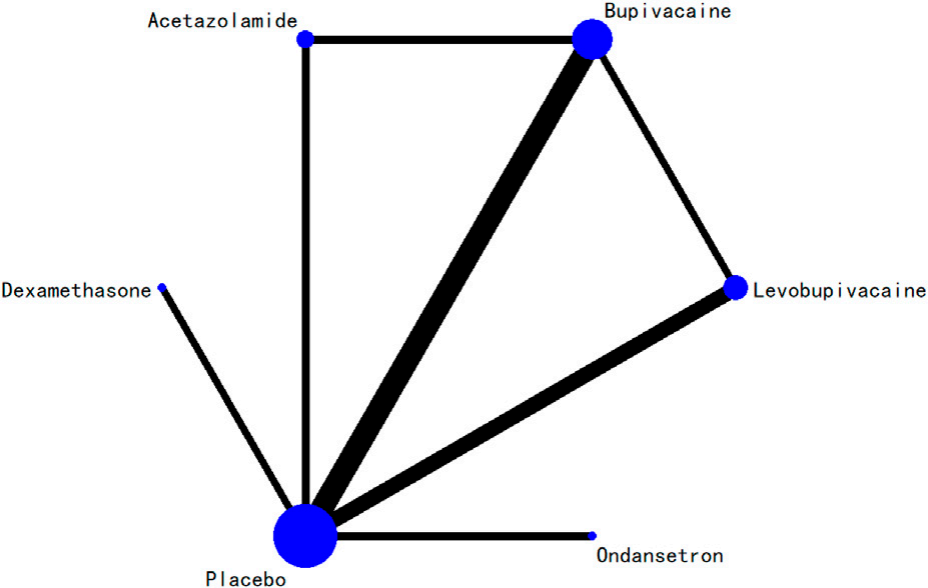
Network evidence diagram for 12-h postoperative visual analog scale score.

**FIGURE 5:**
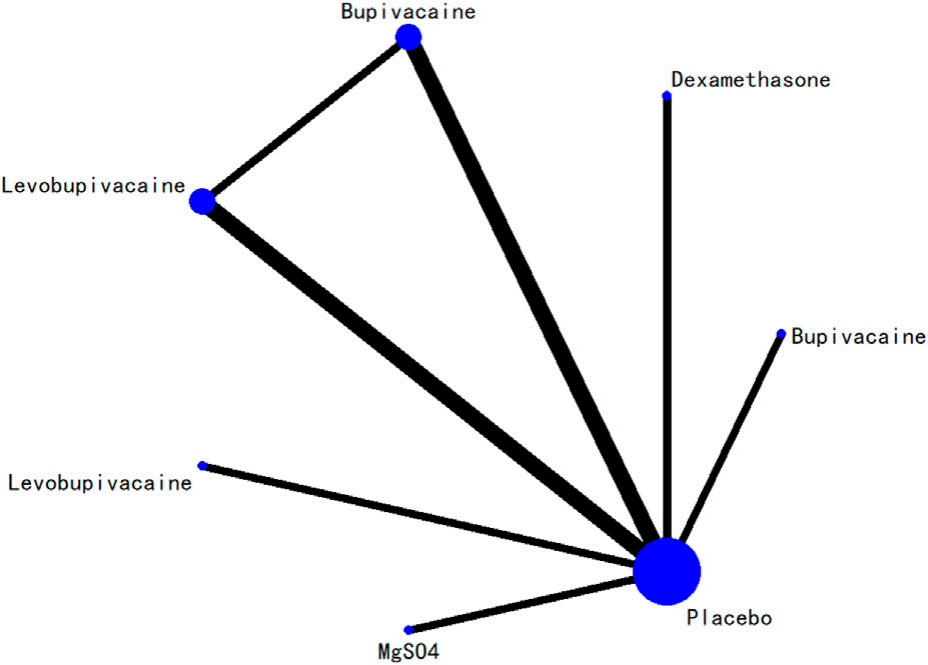
Network evidence diagram for 6-h postoperative visual analog scale score.

**FIGURE 6:**
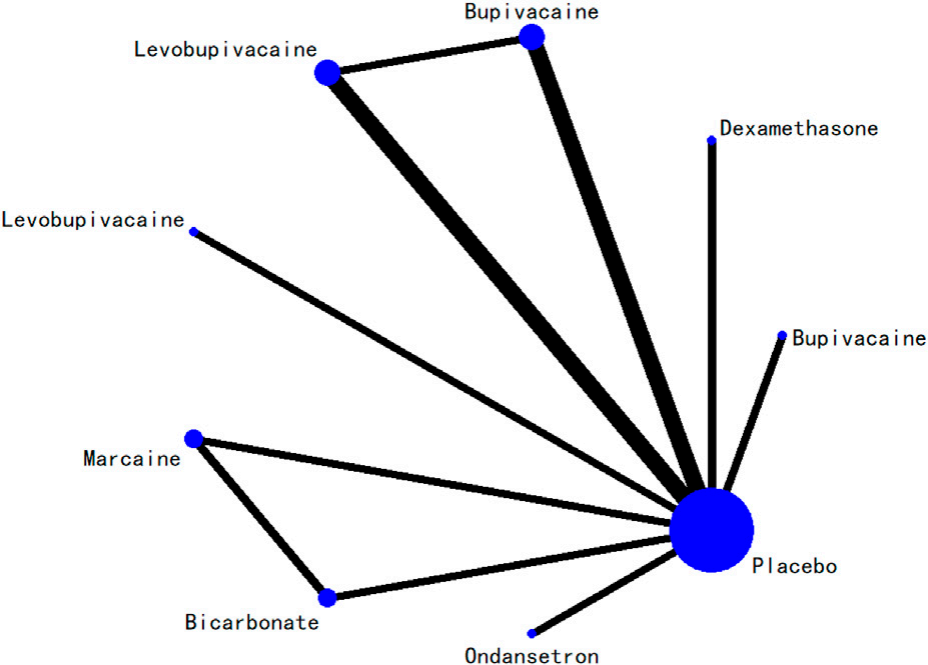
Network evidence diagram for 2-h postoperative visual analog scale score.

**FIGURE 7:**
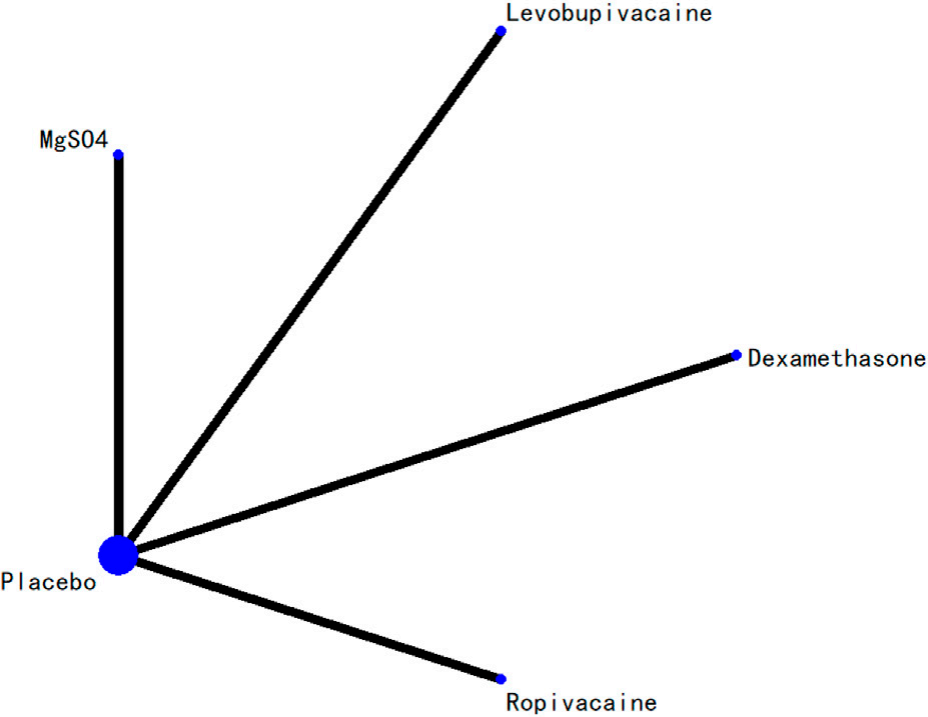
Network evidence diagram for analgesics consumption.

**FIGURE 8:**
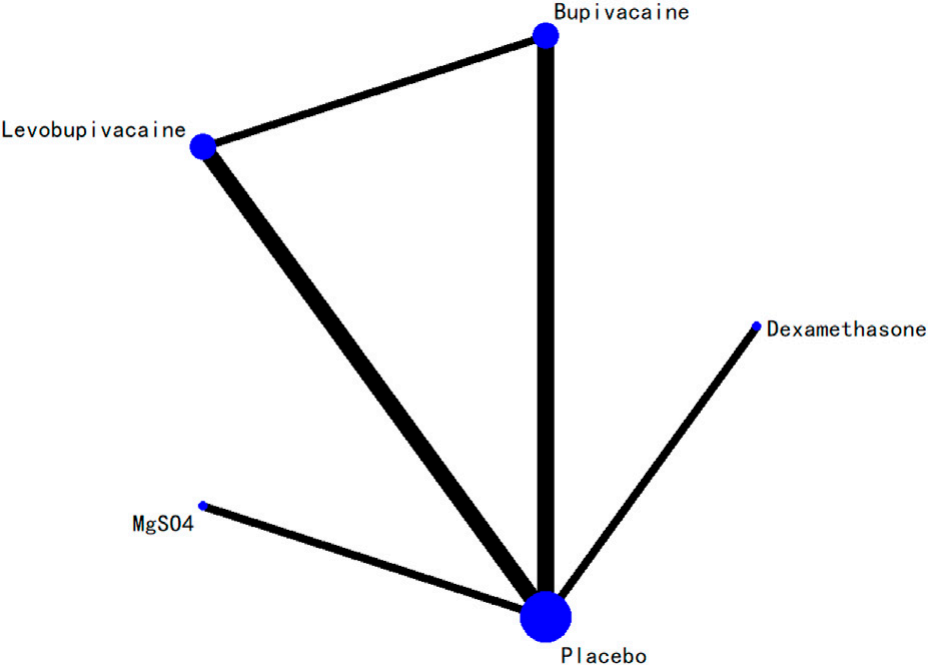
Network evidence diagram for first analgesic requirement time.

The network relationships among all interventions included in the analysis are presented in Figures 3–8. Each node represents a treatment arm. The width of each edge is proportional to the number of trials comparing the two connected interventions. Thicker lines indicate more direct comparative evidence, while thinner lines represent fewer studies. The absence of a connecting line indicates that no direct comparisons were available between those treatments; however, indirect comparisons could be estimated through the network meta-analysis.

### 3.6 Inconsistency test

Since the network for analgesic consumption did not contain any closed loops, an inconsistency test was not applicable. For all other outcomes, closed loops were present. Node-splitting analysis was performed for each loop, and all yielded *p* > 0.05, indicating no significant inconsistency between direct and indirect evidence within the network.

### 3.7 Network meta-analysis results

#### 3.7.1 24-h postoperative visual analog scale score

**FIGURE 9:**
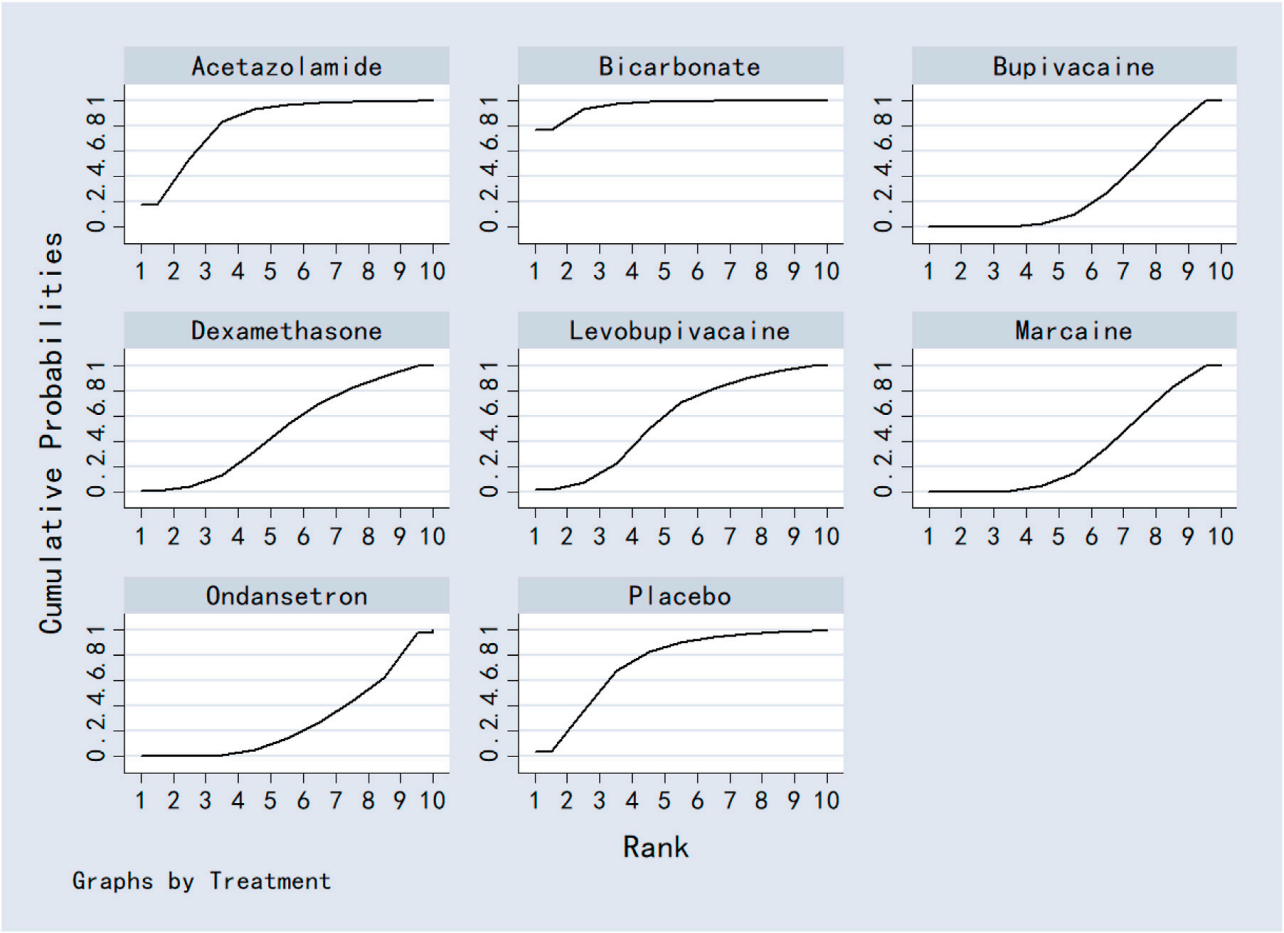
The probability ranking for 24-h postoperative visual analog scale score.

Bicarbonate was associated with significantly lower VAS scores at 24 h compared to dexamethasone, bupivacaine, ondansetron and marcaine. Acetazolamide also outperformed bupivacaine, ondansetron, and marcaine. No other significant differences were observed between interventions. Detailed results were shown in Table 2. Ranking based on SUCRA values was as follows: bicarbonate (96.5%) > acetazolamide (82.7%) > placebo (74.6%) > levobupivacaine (57.7%) > dexamethasone (49.7%) > marcaine (33.2%) > bupivacaine (30.1%) > ondansetron (28.1%) (Figure 9; Table 8).

**TABLE 2:** Network meta-analysis of 24-h postoperative visual analog scale score.

Bicarbonate							
−0.40 (−1.32,0.52)	Acetazolamide						
−0.52 (−1.17,0.12)	−0.12 (−1.02,0.77)	Placebo					
−0.83 (−1.72,0.05)	−0.43 (−1.23,0.36)	−0.31 (−1.16,0.55)	Levobupivacaine				
**−0.94 (−1.83,−0.05)**	−0.54 (−1.34,0.26)	−0.42 (−1.28,0.44)	−0.11 (−0.87,0.65)	Dexamethasone			
**−1.14 (−1.92,−0.35)**	**−0.74 (−1.40,−0.08)**	−0.61 (−1.36,0.14)	−0.30 (−0.93,0.32)	−0.20 (−0.83,0.44)	Marcaine		
**−1.16 (−1.93,−0.39)**	**−0.76 (−1.35,−0.17)**	−0.64 (−1.37,0.09)	−0.33 (−0.95,0.28)	−0.22 (−0.85,0.40)	−0.03 (−0.42,0.36)	Bupivacaine	
**−1.21 (−2.05,−0.36)**	**−0.81 (−1.56,−0.06)**	−0.68 (−1.50,0.13)	−0.38 (−1.08,0.33)	−0.27 (−0.98,0.45)	−0.07 (−0.65,0.50)	−0.04 (−0.60,0.51)	Ondansetron

Bold font indicates that the difference was statistically significant.

#### 3.7.2 12-h postoperative visual analog scale score

**FIGURE 10:**
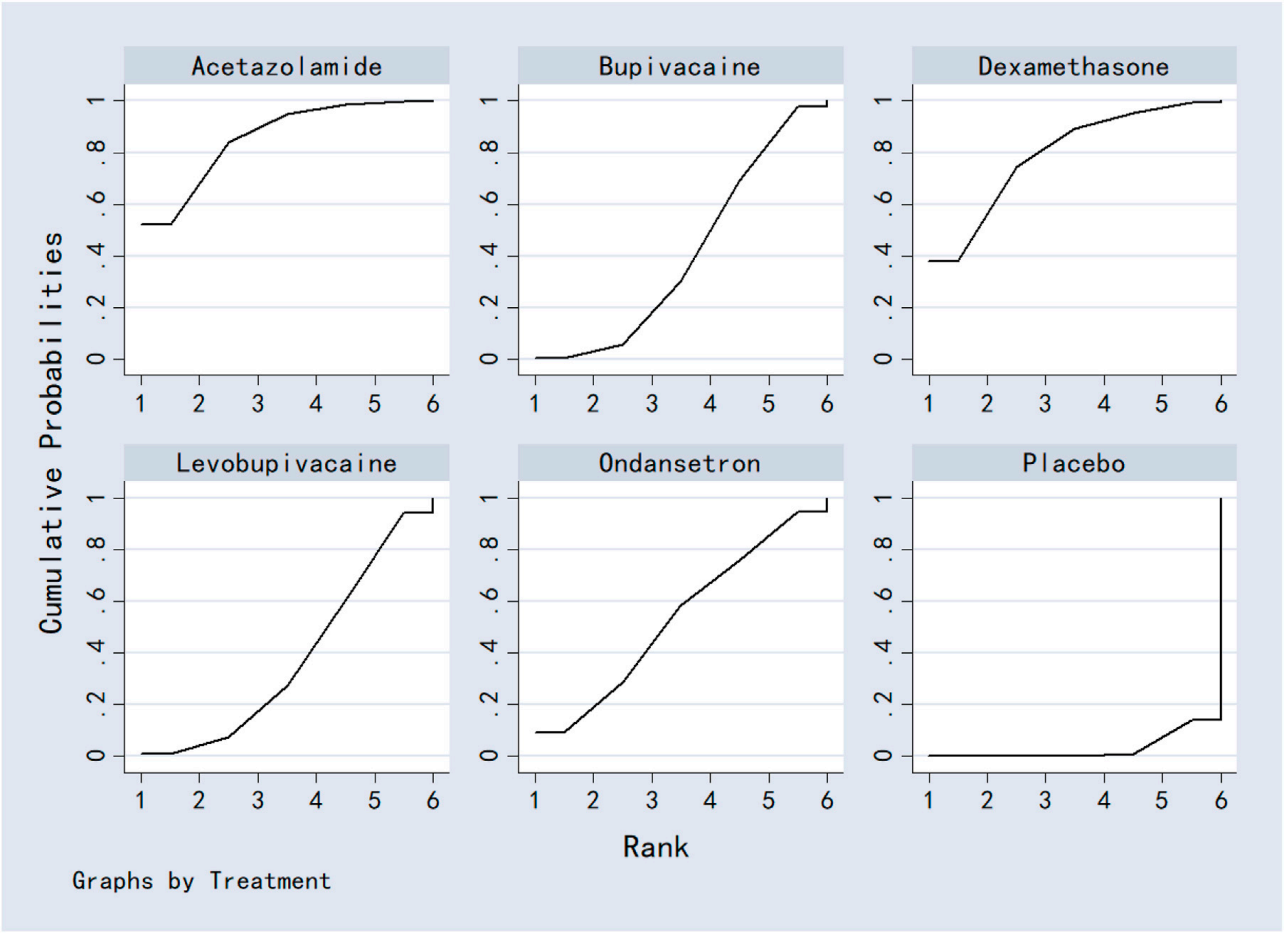
The probability ranking for 12-h postoperative visual analog scale score.

Acetazolamide, dexamethasone, and bupivacaine were significantly superior to placebo in reducing VAS scores at 12 h post-surgery. No other comparisons reached statistical significance. Full results are provided in Table 3. SUCRA rankings were: acetazolamide (85.9%) > dexamethasone (79.3%) > ondansetron (53.2%) > bupivacaine (40.7%) > levobupivacaine (38.0%) > placebo (3.0%) (Figure 10; Table 8).

**TABLE 3:** Network meta-analysis of 12-h postoperative visual analog scale score.

Acetazolamide					
−0.12 (−1.44,1.19)	Dexamethasone				
−0.61 (−1.93,0.71)	−0.49 (−1.83,0.86)	Ondansetron			
−0.81 (−1.73,0.11)	−0.69 (−1.77,0.40)	−0.20 (−1.29,0.89)	Bupivacaine		
−0.85 (−1.91,0.21)	−0.73 (−1.85,0.40)	−0.24 (−1.38,0.90)	−0.04 (−0.75,0.67)	Levobupivacaine	
**−1.35 (−2.27,−0.44)**	**−1.23 (−2.17,−0.29)**	−0.74 (−1.70,0.21)	**−0.54 (−1.08,−0.01)**	−0.50 (−1.12,0.11)	Placebo

Bold font indicates that the difference was statistically significant.

#### 3.7.3 6-h postoperative visual analog scale score

**FIGURE 11:**
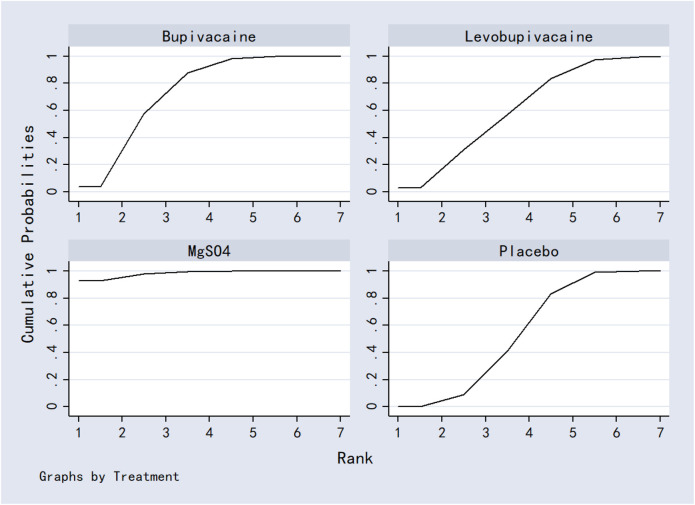
The probability ranking for 6-h postoperative visual analog scale score.

MgSO_4_ was significantly more effective than placebo in reducing VAS scores at 6 h. No other significant differences were detected. Results are shown in Table 4. SUCRA values ranked the interventions as: MgSO_4_ (98.4%) > bupivacaine (74.6%) > levobupivacaine (62.0%) > placebo (55.5%) (Figure 11; Table 8).

**TABLE 4:** Network meta-analysis of 6-h postoperative visual analog scale score.

MgSO4			
−0.78 (−1.67,0.12)	Bupivacaine		
−0.96 (−2.00,0.07)	−0.19 (−1.04,0.67)	Levobupivacaine	
**−1.06 (−1.94,−0.18)**	−0.29 (−0.84,0.27)	−0.10 (−0.94,0.74)	Placebo

Bold font indicates that the difference was statistically significant.

#### 3.7.4 2-h postoperative visual analog scale score

**FIGURE 12:**
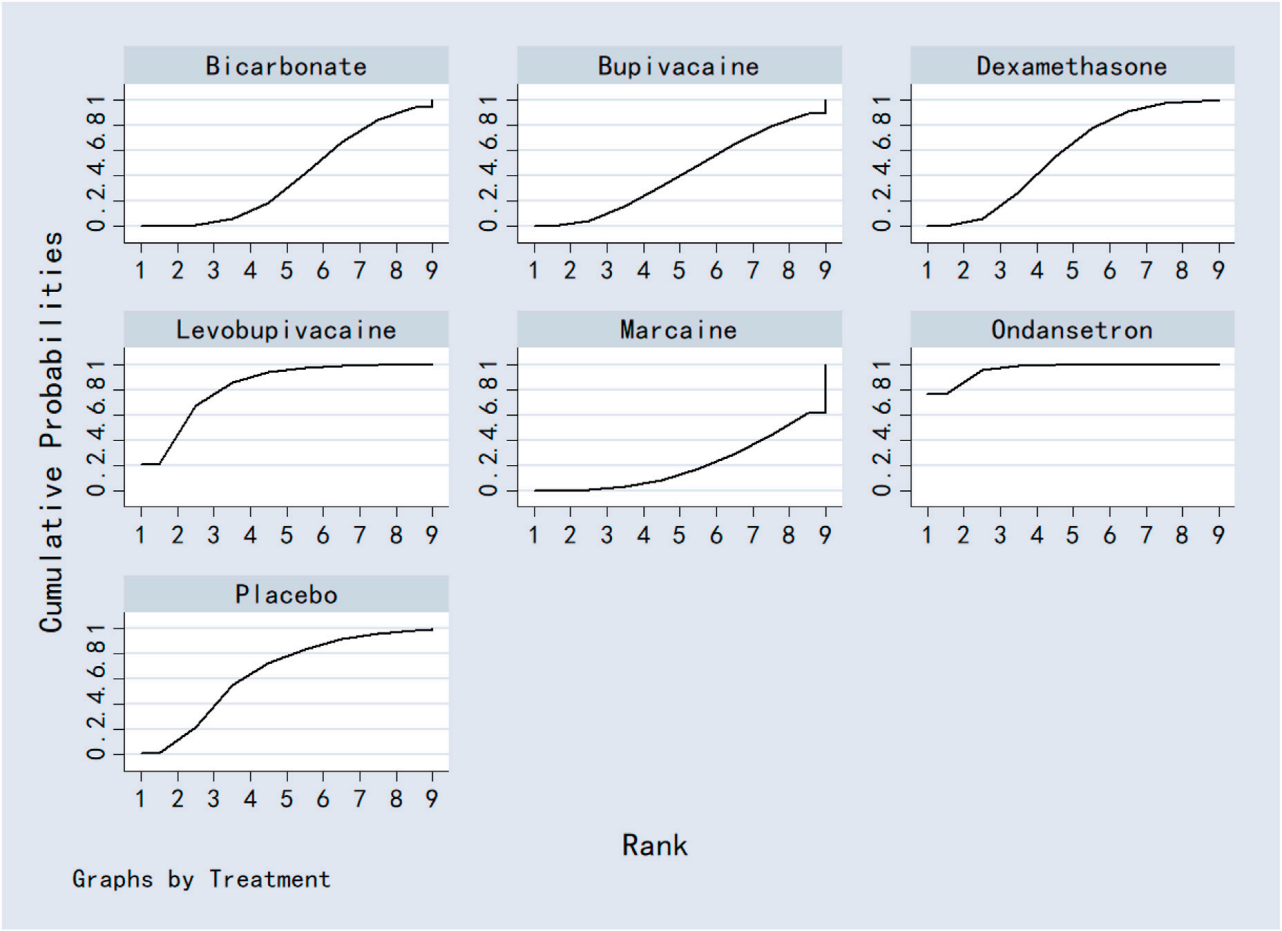
The probability ranking for 2-h postoperative visual analog scale score.

Levobupivacaine resulted in significantly lower VAS scores at 2 h compared to dexamethasone, bupivacaine, bicarbonate, and marcaine. Both dexamethasone and marcaine were less effective than placebo. Other comparisons did not show significant differences. See Table 5 for complete results. SUCRA ranking was: ondansetron (96.4%) > levobupivacaine (83.0%) > placebo (65.2%) > dexamethasone (56.8%) > bupivacaine (41.9%) > bicarbonate (39.1%) > marcaine (20.5%) (Figure 12; Table 8).

**TABLE 5:** Network meta-analysis of 2-h postoperative visual analog scale score.

Ondansetron						
−0.86 (−6.28,4.57)	Levobupivacaine					
1.41 (−6.41,9.22)	0.76 (−7.24,8.76)	Placebo				
−2.59 (−8.09,2.92)	**−1.73 (−2.55,−0.92)**	**1.27 (0.50,2.05)**	Dexamethasone			
−2.27 (−7.91,3.37)	**−1.41 (−2.27,−0.56)**	−1.08 (−8.94,6.79)	0.32 (−0.43,1.07)	Bupivacaine		
−2.92 (−8.37,2.54)	**−2.06 (−2.87,−1.26)**	0.65 (−7.12,8.43)	−0.33 (−0.84,0.18)	−0.65 (−1.41,0.11)	Bicarbonate	
−2.85 (−8.40,2.69)	**−2.00 (−2.79,−1.21)**	**0.92 (0.20,1.64)**	−0.27 (−0.95,0.42)	−0.58 (−1.29,0.13)	0.07 (−0.62,0.76)	Marcaine

Bold font indicates that the difference was statistically significant.

#### 3.7.5 Analgesics consumption

**FIGURE 13:**
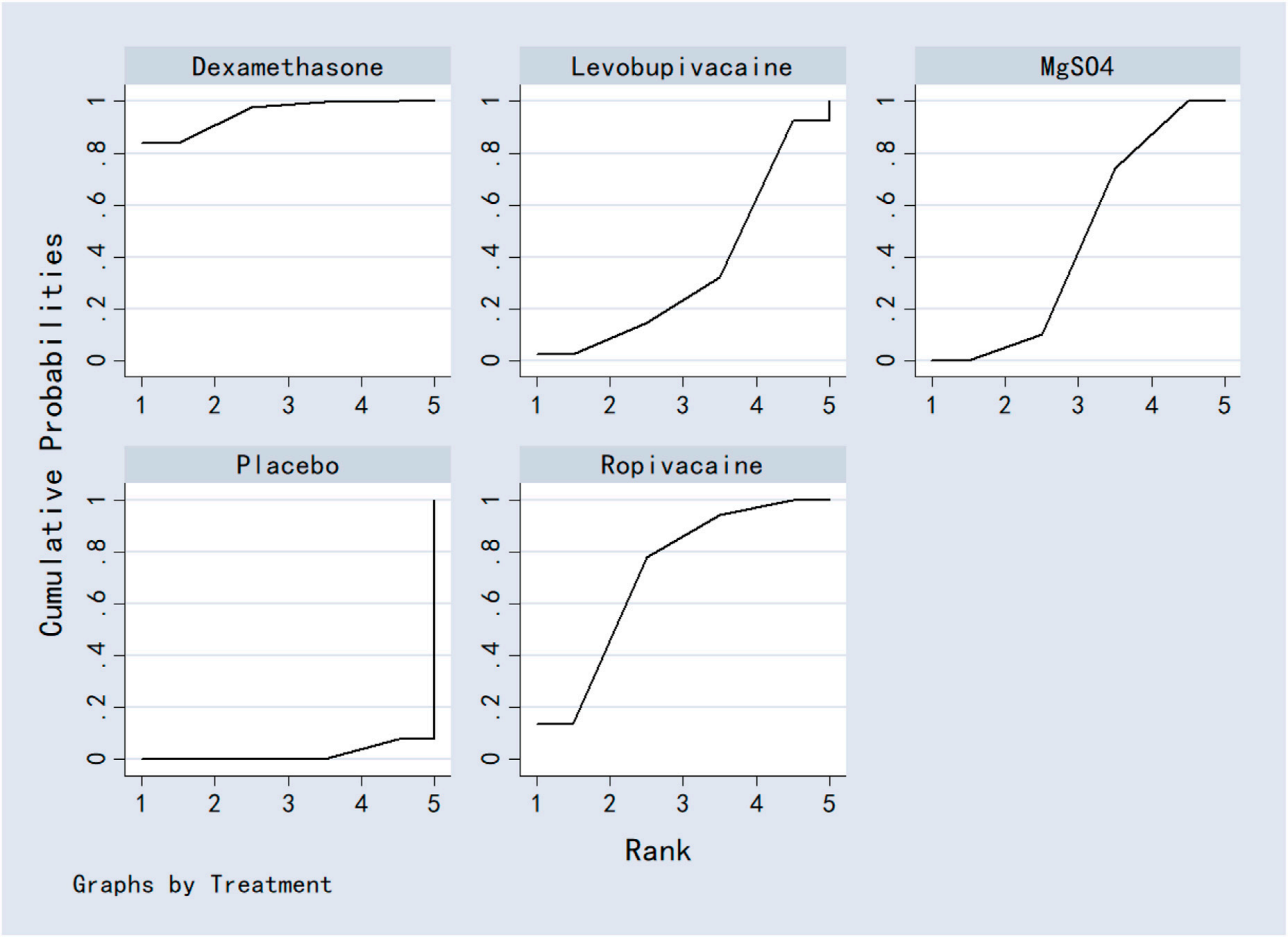
The probability ranking for analgesics consumption.

Dexamethasone was superior to both MgSO_4_ and placebo in reducing analgesic consumption. Ropivacaine and MgSO_4_ were also more effective than placebo. No other comparisons were statistically significant. Results are presented in Table 6. SUCRA values ranked the interventions as: dexamethasone (95.3%) > ropivacaine (71.3%) > MgSO_4_ (46.1%) > levobupivacaine (35.4%) > placebo (1.9%) (Figure 13; Table 8).

**TABLE 6:** Network meta-analysis of analgesics consumption.

Dexamethasone				
−0.41 (−1.18,0.35)	Ropivacaine			
**−0.69 (−1.26,-0.12)**	−0.28 (−0.77,0.21)	MgSO4		
−0.89 (−1.87,0.08)	−0.48 (−1.40,0.44)	−0.20 (−0.98,0.57)	Levobupivacaine	
**−1.58 (−2.35,-0.81)**	**−1.17 (−1.87,−0.47)**	**−0.89 (−1.38,−0.39)**	−0.69 (−1.60,0.23)	Placebo

Bold font indicates that the difference was statistically significant.

#### 3.7.6 First analgesic requirement time

**FIGURE 14:**
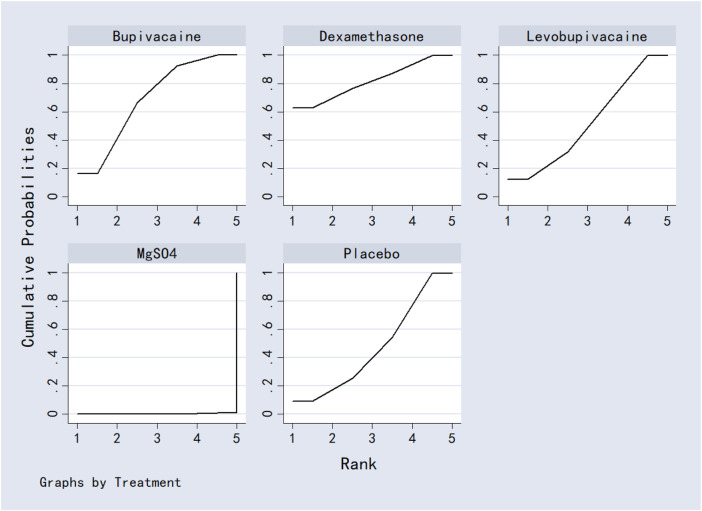
The probability ranking for first analgesic requirement time.

Dexamethasone, bupivacaine, levobupivacaine, and placebo were all associated with a significantly longer time to first analgesic request compared to MgSO_4_. No other significant differences were observed. See Table 7 for details. Based on SUCRA, rankings were: dexamethasone (81.5%) > bupivacaine (68.8%) > levobupivacaine (52.4%) > placebo (47.2%) > MgSO_4_ (0.3%) (Figure 14; Table 8).

**TABLE 7:** Network meta-analysis of first analgesic requirement time.

Dexamethasone				
1.68 (−4.51,7.88)	Bupivacaine			
2.82 (−4.68,10.32)	1.14 (−3.09,5.36)	Levobupivacaine		
3.16 (−4.35,10.67)	1.48 (−2.77,5.72)	0.34 (−4.72,5.40)	Placebo	
**13.94 (4.90,22.99)**	**12.26 (5.67,18.85)**	**11.12 (3.29,18.96)**	**10.78 (2.94,18.63)**	MgSO4

Bold font indicates that the difference was statistically significant.

**TABLE 8:** The surface under the cumulative ranking curve probability ranking.

Interventions	2 h (VAS score)		6 h (VAS score)		12 h (VAS score)		24 h (VAS score)		Consumption of analgesics		First analgesic requirement time	
	SUCRA%	Ranking	SUCRA%	Ranking	SUCRA%	Ranking	SUCRA%	Ranking	SUCRA%	Ranking	SUCRA%	Ranking
Acetazolamide	NA	NA	NA	NA	**85.9**	**1**	82.7	2	NA	NA	NA	NA
Bicarbonate	39.1	6	NA	NA	NA	NA	**96.5**	**1**	NA	NA	NA	NA
Bupivacaine	41.9	5	74.6	2	40.7	4	30.1	7	NA	NA	68.8	2
Dexamethasone	56.8	4	NA	NA	79.3	2	49.7	5	**95.3**	**1**	**81.5**	**1**
Levobupivacaine	83	2	62	3	38	5	57.7	4	35.4	4	52.4	3
Marcaine	20.5	7	NA	NA	NA	NA	33.2	6	NA	NA	NA	NA
MgSO4	NA	NA	**98.4**	**1**	NA	NA	NA	NA	46.1	3	0.3	5
Ondansetron	**96.4**	**1**	NA	NA	53.2	3	28.1	8	NA	NA	NA	NA
Placebo	65.2	3	55.5	4	3	6	74.6	3	1.9	5	47.2	4
Ropivacaine	NA	NA	NA	NA	NA	NA	NA	NA	71.3	2	NA	NA

NA, data Not Available; SUCRA, the surface under the cumulative ranking curve; VAS, visual analog scale.

Bold font indicates that the difference was statistically significant.

### 3.8 Publication bias

**FIGURE 15:**
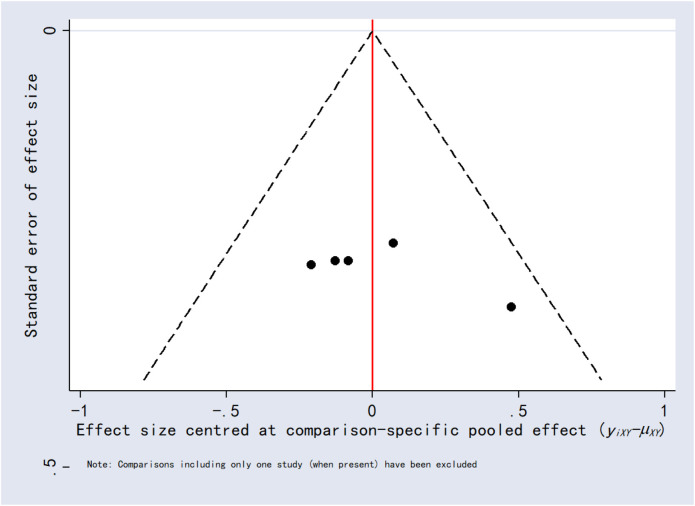
Funnel plot for 24-h postoperative visual analog scale score.

**FIGURE 16:**
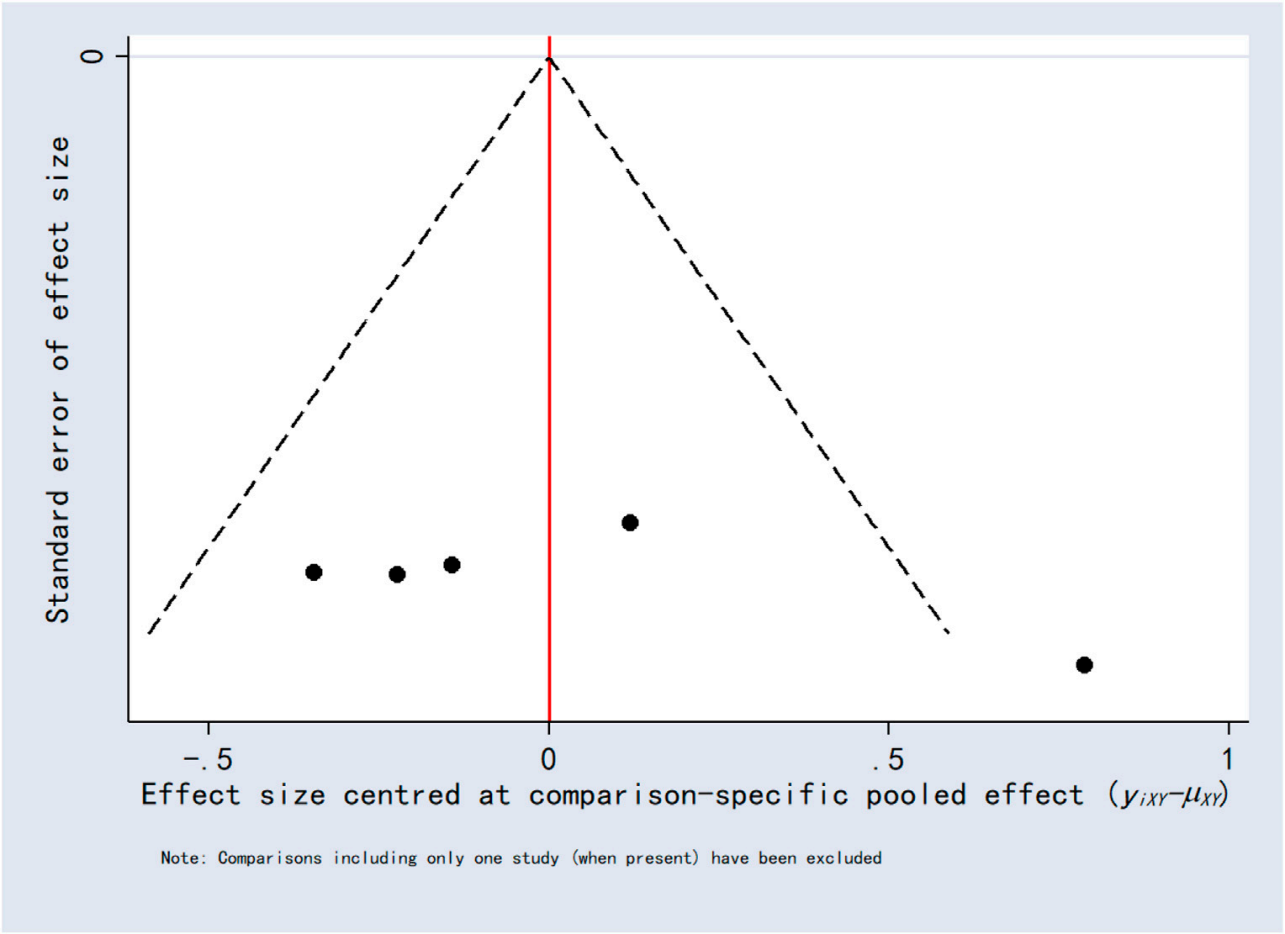
Funnel plot for 12-h postoperative visual analog scale score.

**FIGURE 17:**
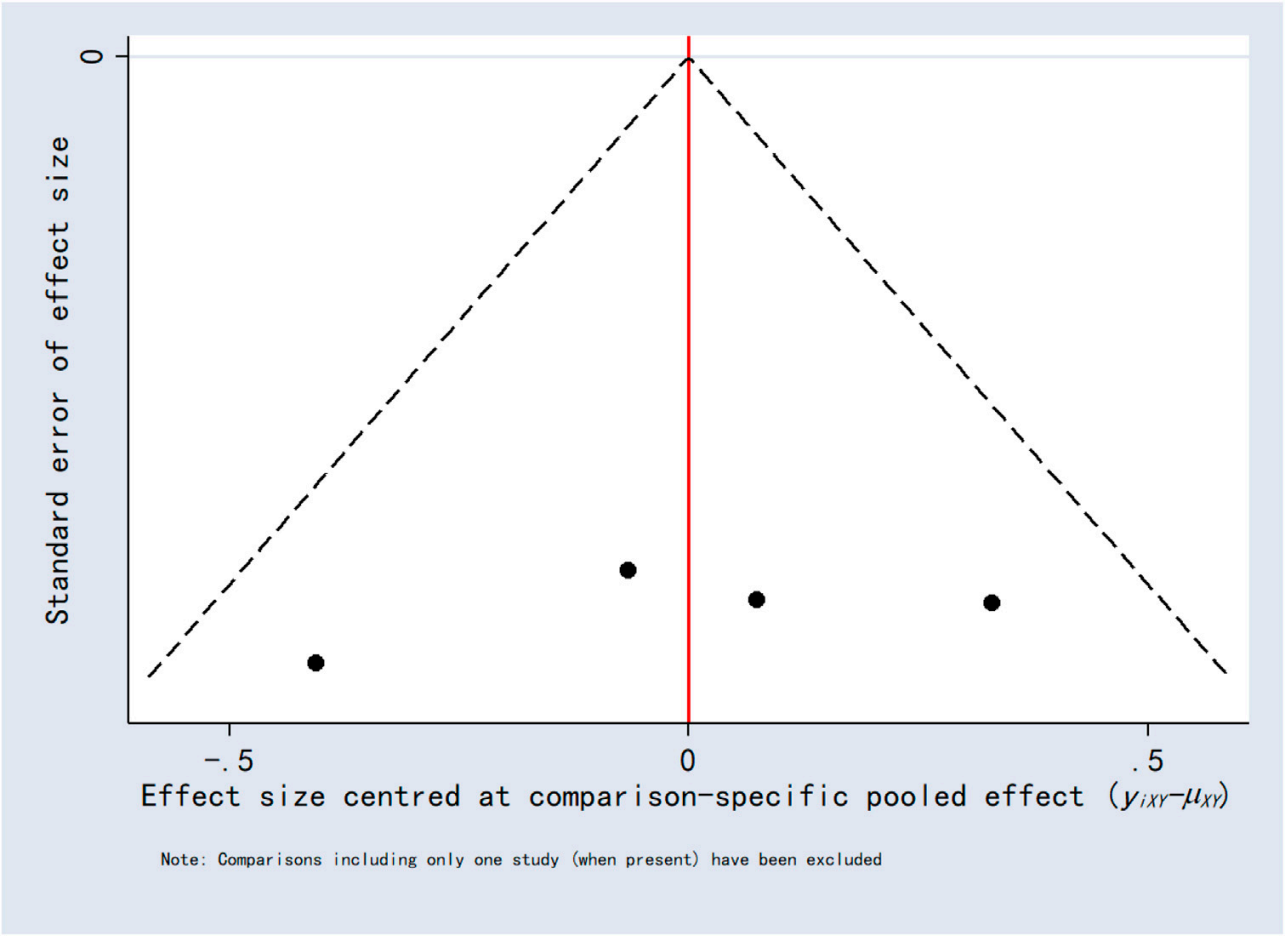
Funnel plot for 6-h postoperative visual analog scale score.

**FIGURE 18:**
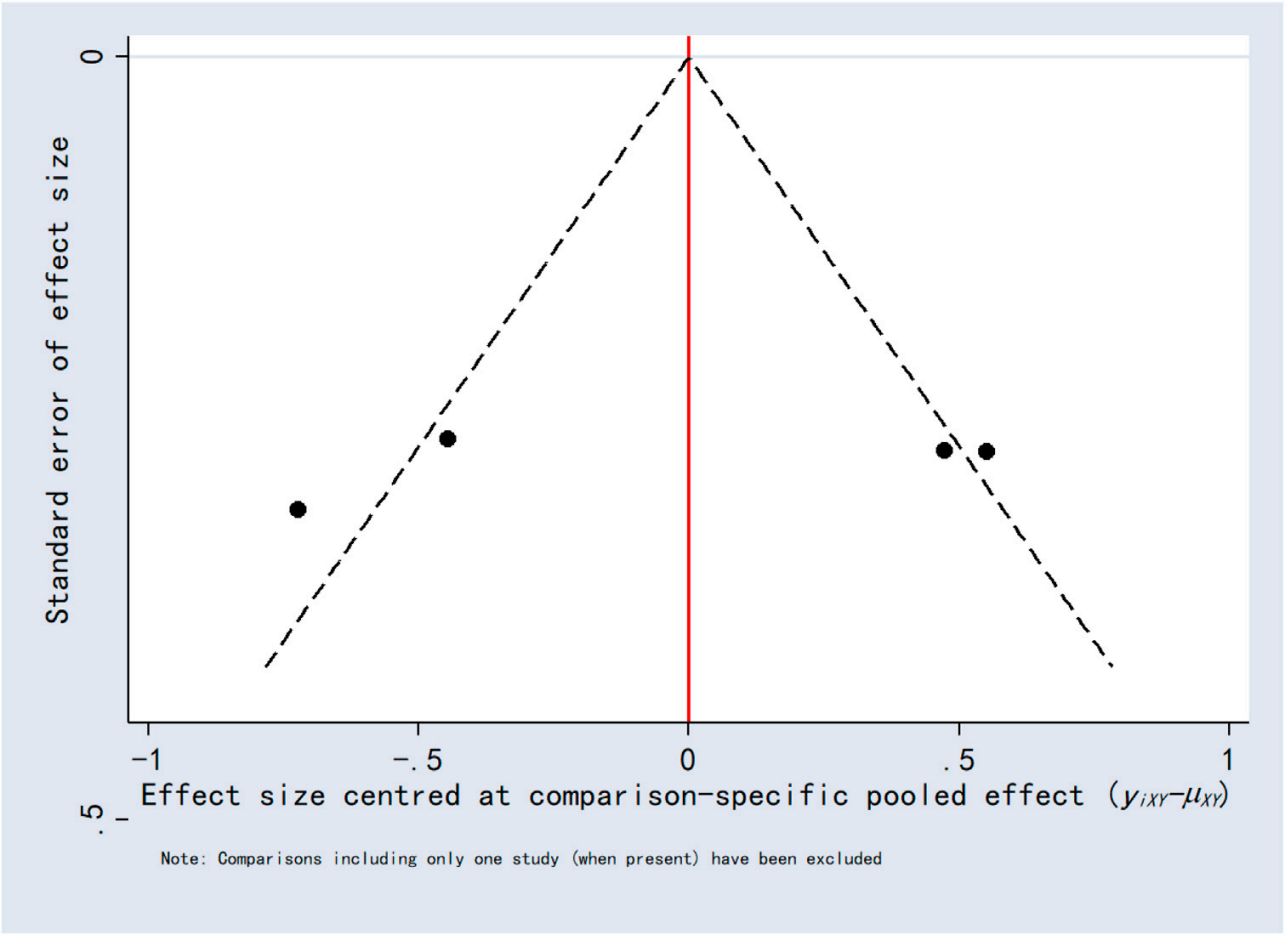
Funnel plot for 2-h postoperative visual analog scale score.

**FIGURE 19:**
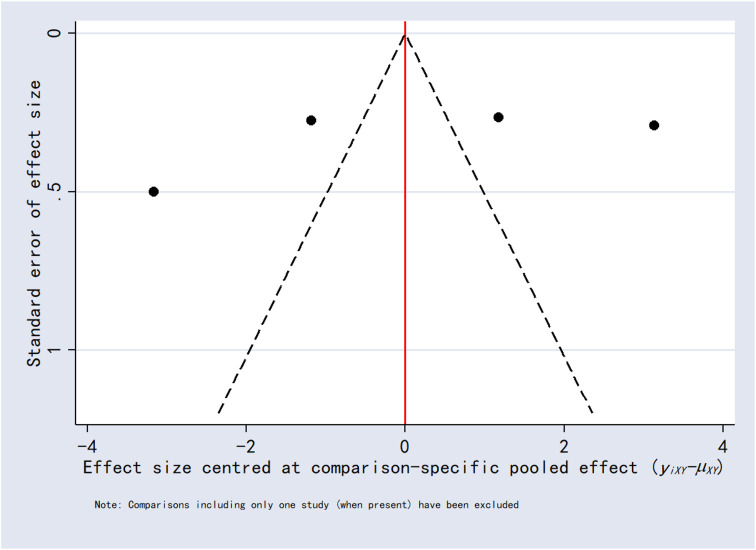
Funnel plot for analgesics consumption.

**FIGURE 20:**
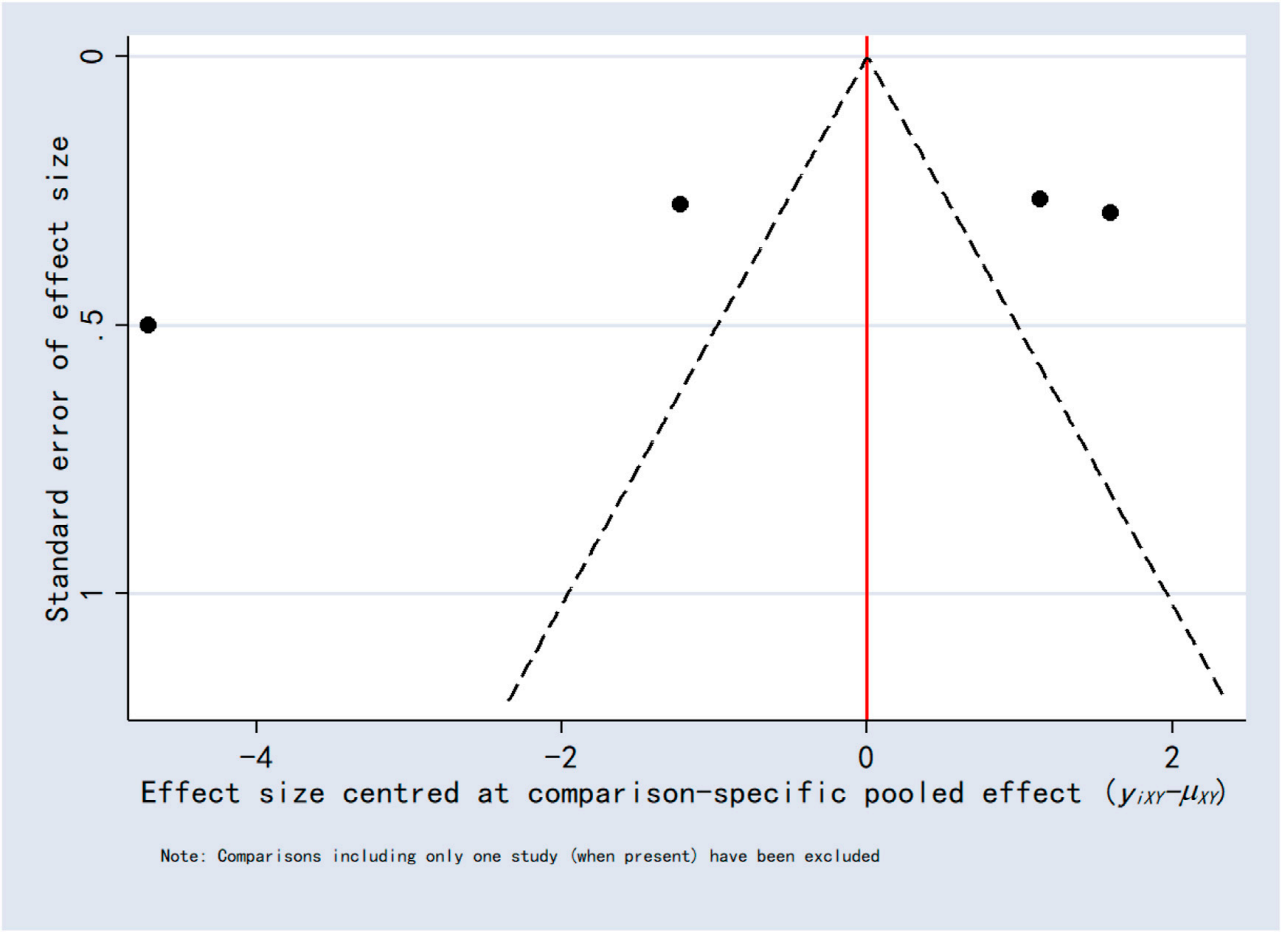
Funnel plot for first analgesic requirement time.

Funnel plots for the outcomes exhibited asymmetric distributions, suggesting the possible presence of publication bias or small-study effects (Figures 15–20).

## 4 Discussion

In this network meta-analysis of eleven RCTs involving 667 patients undergoing LC under general anesthesia, we comprehensively evaluated and ranked ten different interventions for postoperative analgesia. The results demonstrated that bicarbonate was most effective in reducing VAS scores at 24 h post-surgery, acetazolamide at 12 h, MgSO_4_ at 6 h, and ondansetron at 2 h. Dexamethasone was associated with the lowest analgesic consumption and the longest time to first analgesic request. These findings hold considerable clinical relevance, offering evidence-based guidance for drug selection in post-LC pain management.

A multimodal analgesia regimen combining non-opioid and opioid agents—such as local anesthetics and non-steroidal anti-inflammatory drugs—is commonly employed for postoperative pain control (Jiang and Ye, 2022). Among local anesthetics, ropivacaine is known for its longer duration of action and more favorable safety profile compared to bupivacaine (Das and Deshpande, 2017). Acetazolamide, a carbonic anhydrase inhibitor, mitigates pain by reducing intra-abdominal acidity (Moazeni et al., 2010). Our analysis ranked it second for 24-h analgesia and first for pain reduction at 12 h, underscoring its potential utility in clinical practice.

LC is a common surgical procedure that involves insufflating the abdominal cavity with gas (most commonly carbon dioxide) to create space and improve the surgeon’s visual field during the operation. However, this gas insufflation increases intra-abdominal pressure and stimulates the production of acidic metabolites in abdominal tissues, leading to heightened acidity. As an alkaline agent, bicarbonate can counteract this gas-induced acidity during laparoscopic cholecystectomy, potentially reducing postoperative pain (Li et al., 2021; Mahmoud et al., 2022). Consistent with this, bicarbonate emerged as the top-ranked intervention for 24-h VAS scores. Nikoubakht et al. (2022) also reported comparable efficacy between bicarbonate and local anesthetics in reducing postoperative pain.

MgSO4 was administered intraperitoneally to modulate the hemodynamic stress response induced by pneumoperitoneum and to reduce postoperative pain (Ali et al., 2015). Magnesium exerts its analgesic effect by blocking NMDA receptors, which play a key role in neuronal signaling and pain perception (Ryu et al., 2008; Sirvinskas and Laurinaitis, 2002). Our network meta-analysis identified MgSO_4_ as the most effective agent for pain control at 6 h. Previous clinical trials have shown that patients with LC who received intraperitoneal MgSO4 exhibited significantly lower postoperative pain scores and reduced opioid consumption compared to those receiving intravenous instillation (Elfiky et al., 2018). Sravanthi et al. (Sravanthi et al., 2023) further confirmed that MgSO_4_ reduces pain and vomiting without increasing side effects.

Multiple studies have demonstrated that 5-HT3-antagonists possess anti-inflammatory and analgesic properties, indicating their potential clinical role in pain management (Färber et al., 2001; Papadopoulos et al., 2000; Louca et al., 2016). Ondansetron has been shown to effectively alleviate the local pain associated with propofol injection, with an efficacy comparable to that of lidocaine (Pei et al., 2017). Furthermore, evidence suggests that the local anesthetic effect of ondansetron is approximately five times more potent than that of lidocaine (Abdelaziz et al., 2021). The precise mechanism underlying its local anesthetic action remains incompletely understood. However, it may involve the blockade of sodium channels and peripheral 5-HT3 receptors implicated in pain signaling pathways (Wasinwong et al., 2022). Abdelaziz et al. (Abdelaziz et al., 2021) demonstrated that intraperitoneal ondansetron reduces pain and reduce the frequency of nausea and vomiting in LC patients. Our findings further support its strong performance in early analgesia (2-h VAS), highlighting its research potential.

Dexamethasone injection, a corticosteroid with potent anti-inflammatory properties, effectively mitigates the inflammatory response associated with postoperative tissue injury and reduces peripheral pain sensitization. This action prolongs the duration of peripheral nerve blockade, thereby enhancing analgesia (Abdelhedi et al., 2023; Jamil and Qaisar, 2022; Nazemroaya et al., 2022). Intraperitoneal instillation of dexamethasone has been shown to improve postoperative pain control by alleviating abdominal and scapular pain, as well as decreasing the consumption of morphine and other analgesics (Abdelhedi et al., 2023). Asgari et al.’s (Asgari et al., 2012) demonstrated that intraperitoneal instillation of 16 mg dexamethasone significantly reduced pain severity following laparoscopy compared to placebo and potentially reduced the need for anesthetic analgesics. Although dexamethasone did not rank among the most effective agents in lowering VAS scores, it was superior in reducing analgesic consumption and prolonging the time to first analgesic requirement. Therefore, dexamethasone appears to be a promising adjunctive medication for decreasing analgesic use and extending the duration of pain relief.

## 5 Advantages of this study

This study has several strengths. First, it synthesizes a substantial body of evidence from eleven RCTs, encompassing 667 patients undergoing LC. The considerable sample size enhances the statistical power and generalizability of our findings. Second, by incorporating all available direct and indirect evidence, we were able to rank multiple interventions according to their efficacy in reducing postoperative VAS scores at various time points, thereby offering practical guidance for analgesic selection. Lastly, our analysis identifies dexamethasone as a highly effective adjunct for reducing analgesic consumption and prolonging the duration of analgesia, which holds significant implications for clinical practice and postoperative pain management strategies.

## 6 Limitation

This study also has several limitations. First, the amount of direct evidence for certain interventions remains limited. The relatively small number of studies included for each outcome may increase the risk of selective reporting bias or small-study effects, which could affect the statistical robustness and reliability of the meta-analytic results. Therefore, these findings should be interpreted with caution. Second, the results are derived from aggregated data, and variations in drug dosages, timing of instillation of drugs, surgical techniques, and methods of pain assessment across studies may introduce clinical and methodological heterogeneity. Finally, most studies assessed pain outcomes only within the first 24 h postoperatively, lacking long-term follow-up data on pain control and recovery. Thus, larger and more rigorously designed randomized controlled trials with extended observation periods are needed to validate these findings.

## 7 Conclusion

In summary, bicarbonate, acetazolamide, MgSO_4_, and ondansetron each demonstrate distinct analgesic profiles at different time points following LC. Dexamethasone appears to be a promising adjunctive agent for reducing analgesic requirements and extending the duration of analgesia. However, given the limitations of the currently available evidence, these conclusions should be further verified through larger, high-quality studies.

## Data Availability

The datasets presented in this study can be found in online repositories. The names of the repository/repositories and accession number(s) can be found in the article/Supplementary Material.
