# Supplementary material for: The effect of intraperitoneal instillation of drugs on postoperative analgesia after laparoscopic cholecystectomy: a network meta-analysis
Source: Front Pharmacol. 2025 Sep 12;16:1646917. doi: 10.3389/fphar.2025.1646917 (PMC12463911; doi:10.3389/fphar.2025.1646917)
Supplement: Supplementary file 1 [file Supplementaryfile1.docx]

| **Supplementary Material Table S1.Search strategy** |
| --- |
| ((("Cholecystectomy, Laparoscopic"[Mesh] OR "Laparoscopic Cholecystectomy"[tiab] OR "LC"[tiab]) AND ("Pain, Postoperative"[Mesh] OR "Postoperative Pain"[tiab] OR "Post-operative Pain"[tiab] OR "Postoperative Analgesia"[tiab] OR "Analgesia"[Mesh] OR "Analgesics"[Mesh])) AND ("Injections, Intraperitoneal"[Mesh] OR "Intraperitoneal"[tiab] OR "IP"[tiab] OR "Instillation"[tiab] OR "Peritoneal Lavage"[Mesh] OR "Lavage"[tiab])) AND ("Randomized Controlled Trial"[Publication Type] OR "Randomized Controlled Trials as Topic"[Mesh] OR "randomized"[tiab] OR "randomised"[tiab] OR "placebo"[tiab] OR "clinical trials as topic"[Mesh] OR "clinical trial"[tiab] OR "RCT"[tiab]) |

| **Supplementary Material Table S2.The evaluation results of the bias risks for each of the included trials** | | | | | | | |
| --- | --- | --- | --- | --- | --- | --- | --- |
| Study, year | Random sequence generation | Allocation concealment | Blinding of participants and personnel | Blinding of outcome assessment | Incomplete outcome data | Selective Reporting | Other bias |
| Abdelhedi et al 2023 | Low risk | Low risk | Low risk | Low risk | Low risk | Low risk | Low risk |
| Labaille et al 2002 | Unclear | Unclear | Low risk | Low risk | Low risk | Low risk | Low risk |
| Honca et al 2014 | Low risk | Unclear | Low risk | Low risk | Low risk | Low risk | Low risk |
| Melidi et al 2016 | Low risk | Unclear | Low risk | Low risk | Low risk | Low risk | Low risk |
| Nikoubakht et al 2022 | Unclear | Unclear | Low risk | Low risk | Low risk | Low risk | Low risk |
| Putta et al 2019 | Low risk | Low risk | Low risk | Low risk | Low risk | Low risk | Low risk |
| Rahimzadeh et al 2018 | Low risk | Unclear | Low risk | Low risk | Low risk | Low risk | Low risk |
| Vijayaraghavalu et al 2021 | Low risk | Unclear | Low risk | Low risk | Low risk | Low risk | Low risk |
| Sravanthi et al 2023 | Low risk | Low risk | Low risk | Low risk | Low risk | Low risk | Low risk |
| Beder et al 2018 | Low risk | Unclear | Low risk | Low risk | Low risk | Low risk | Low risk |
| Abdelaziz et al 2021 | Low risk | Low risk | Low risk | Low risk | Low risk | Low risk | Low risk |

| **Supplementary Material Table S3. The results of the direct comparison meta-analysis** | | | | | | | |
| --- | --- | --- | --- | --- | --- | --- | --- |
| Outcomes | Intervention | The number of studies | Heterogeneity | | Model | Pooled analysis results | |
|  |  |  | *I²* | *P*-value |  | SMD(95%Cl) | *P*-value |
| 24-hour postoperative Visual Analog Scale score | Bupivacaine vs Placebo | 1 | NA | NA | NA | **-0.773(-1.317,-0.229)** | **0.005** |
|  | Dexamethasone vs Placebo | 1 | NA | NA | NA | **-0.883(-1.414,-0.352)** | **0.001** |
|  | Acetazolamide vs Bupivacaine | 1 | NA | NA | NA | **-1.003(-1.663,-0.344)** | **0.003** |
|  | Acetazolamide vs Placebo | 1 | NA | NA | NA | **-1.370(-2.062,-0.678)** | **< 0.00001** |
|  | Bicarbonate vs Marcaine | 1 | NA | NA | NA | -0.629(-1.282,0.023) | 0.059 |
|  | Bicarbonate vs Placebo | 1 | NA | NA | NA | **-1.483(-2.196,-0.770)** | **< 0.00001** |
|  | Bupivacaine vs Levobupivacaine | 1 | NA | NA | NA | 0(-0.506,0.506) | 1.000 |
|  | Bupivacaine vs Placebo | 3 | 47.1% | 0.151 | Fixed | **-0.59(-0.909,-0.271)** | **< 0.00001** |
|  | Levobupivacaine vs Placebo | 3 | 0 | 0.970 | Fixed | **-0.535( -0.816,-0.255)** | **< 0.00001** |
|  | Marcaine vs Placebo | 1 | NA | NA | NA | **-1.245(-1.934,-0.556)** | **< 0.00001** |
|  | Ondansetron vs Placebo | 1 | NA | NA | NA | **-0.750(-1.324,-0.176)** | **0.010** |
| 12-hour postoperative Visual Analog Scale score | Levobupivacaine vs Placebo | 2 | 0 | 0.595 | Fixed | **-0.562(-0.913,-0.211)** | **0.002** |
|  | Acetazolamide vs Placebo | 1 | NA | NA | NA | **-1.000(-1.659,-0.340)** | **0.003** |
|  | Acetazolamide vs Bupivacaine | 1 | NA | NA | NA | **-1.449(-2.149,-0.749)** | **< 0.00001** |
|  | Bupivacaine vs Levobupivacaine | 1 | NA | NA | NA | -0.232(-0.740,0.276) | 0.371 |
|  | Bupivacaine vs Placebo | 3 | 76.6% | 0.014 | Random | -0.562(-1.230,0.107) | 0.100 |
|  | Dexamethasone vs Placebo | 1 | NA | NA | NA | **-1.248(-1.803,-0.693)** | **< 0.00001** |
|  | Ondansetron vs Placebo | 1 | NA | NA | NA | **-0.74(-1.32, -0.17)** | **0.001** |
| 6-hour postoperative Visual Analog Scale score | Levobupivacaine vs Placebo | 2 | 0 | 0.570 | Fixed | **-0.684(-1.038,-0.329)** | **< 0.00001** |
|  | Bupivacaine vs Levobupivacaine | 1 | NA | NA | NA | -0.143(-0.650,0.364) | 0.581 |
|  | Bupivacaine vs Placebo | 3 | 55.2% | 0.107 | Random | **-1.010(-1.482,-0.537)** | **< 0.00001** |
|  | Dexamethasone vs Placebo | 1 | NA | NA | NA | **-1.85(-2.47, -1.24)** | **< 0.00001** |
|  | Levobupivacaine vs Placebo | 1 | NA | NA | NA | 0.00(-0.46, 0.46) | 1.000 |
|  | MgSO4 vs Placebo | 1 | NA | NA | NA | -0.47(-0.96, 0.03) | 0.070 |
| 2-hour postoperative Visual Analog Scale score | Dexamethasone vs Placebo | 1 | NA | NA | NA | **-2.00(-2.63, -1.37)** | **< 0.00001** |
|  | Levobupivacaine vs Placebo | 1 | NA | NA | NA | 0.00(-0.46, 0.46) | 1.000 |
|  | Bicarbonate vs Marcaine | 1 | NA | NA | NA | **1.55(0.81, 2.28)** | **< 0.0001** |
|  | Bicarbonate vs Placebo | 1 | NA | NA | NA | **-1.19(-1.87, -0.50)** | **0.0007** |
|  | Bupivacaine vs Levobupivacaine | 1 | NA | NA | NA | -0.38(-0.89, 0.14) | 0.150 |
|  | Bupivacaine vs Placebo | 3 | 82.3% | 0.004 | Random | **-0.797(-1.538,-0.055)** | **0.035** |
|  | Levobupivacaine vs Placebo | 2 | 84.9% | 0.010 | Random | -0.405(-1.312,0.502) | 0.382 |
|  | Marcaine vs Placebo | 1 | NA | NA | NA | **-3.17(-4.15, -2.20)** | **< 0.00001** |
|  | Ondansetron vs Placebo | 1 | NA | NA | NA | -0.32(-0.87, 0.24) | 0.270 |
| Analgesics consumption | Dexamethasone vs Placebo | 1 | NA | NA | NA | **-0.69(-1.21, -0.17)** | **0.009** |
|  | Placebo vs Ropivacaine | 1 | NA | NA | NA | -0.20(-0.98, 0.57) | 0.610 |
|  | MgSO4 vs Placebo | 1 | NA | NA | NA | -0.28(-0.77, 0.21) | 0.260 |
|  | Levobupivacaine vs Placebo | 1 | NA | NA | NA | **0.89(0.40, 1.38)** | **0.0004** |
| First analgesic requirement time | MgSO4 vs Placebo | 1 | NA | NA | NA | **1.69(1.11, 2.26)** | **< 0.00001** |
|  | Levobupivacaine vs Placebo | 1 | NA | NA | NA | **-1.56(-2.10, -1.02)** | **< 0.00001** |
|  | Bupivacaine vs Placebo | 1 | NA | NA | NA | **-4.55(-5.53, -3.57)** | **< 0.00001** |
|  | Bupivacaine vs Levobupivacaine | 1 | NA | NA | NA | **0.81(0.28, 1.34)** | **0.003** |
|  | Bupivacaine vs Placebo | 1 | NA | NA | NA | **1.58(1.00, 2.17)** | **< 0.00001** |
|  | Levobupivacaine vs Placebo | 1 | NA | NA | NA | **1.26(0.70, 1.82)** | **< 0.00001** |
|  | Dexamethasone vs Placebo | 1 | NA | NA | NA | **-12.29(-14.62, -9.96)** | **< 0.00001** |
| SMD, standardized mean difference;Cl,confidence interval;NA,data Not Available. | | | | | | | |
